# Anti-CD38 antibody therapy for patients with relapsed/refractory multiple myeloma: differential mechanisms of action and recent clinical trial outcomes

**DOI:** 10.1007/s00277-022-04917-5

**Published:** 2022-08-09

**Authors:** Xavier Leleu, Thomas Martin, Katja Weisel, Fredrik Schjesvold, Shinsuke Iida, Fabio Malavasi, Salomon Manier, Chang-Ki Min, Enrique M. Ocio, Charlotte Pawlyn, Aurore Perrot, Hang Quach, Joshua Richter, Ivan Spicka, Kwee Yong, Paul G. Richardson

**Affiliations:** 1 Service d’Hématologie Et Thérapie Cellulaire, CHU and CIC Inserm 1402, Poitiers Cedex, France; 2 Department of Medicine, University of California at San Francisco, San Francisco, CA USA; 3 University Medical Center Hamburg-Eppendorf (UKE), Hamburg, Germany; 4 Oslo Myeloma Center, Department of Hematology, KG Jebsen Center for B Cell Malignancies, Oslo University Hospital, University of Oslo, Oslo, Norway; 5 Department of Hematology and Oncology, Nagoya City University, Nagoya, Japan; 6 Department of Medical Sciences, University of Torino Medical School, Fondazione Ricerca Molinette, Turin, Italy; 7 Department of Hematology, CHU, Universite de Lille, Lille, France; 8 Department of Hematology, College of Medicine, Catholic Hematology Hospital and Leukemia Research Institute, Seoul St. Mary’s Hospital, The Catholic University of Korea, Seoul, Korea; 9 Hospital Universitario Marqués de Valdecilla (IDIVAL), Universidad de Cantabria, Santander, Spain; 10 Division of Cancer Therapeutics, The Institute of Cancer Research, London, UK; 11 Department of Hematology, Institut Universitaire du Cancer de Toulouse, Toulouse, France; 12 Clinical Haematology Service, St Vincent’s Hospital, University of Melbourne, Melbourne, Australia; 13 Division of Hematology and Medical Oncology, Tisch Cancer Institute, Mount Sinai, New York, NY USA; 14 Department of Medicine, Department of Hematology, First Faculty of Medicine, Charles University and General Hospital, Prague, Czech Republic; 15 Department of Haematology, University College, Hospitals NHS Foundation Trust, London, UK; 16 Department of Medical Oncology, Dana-Farber Cancer Institute, Boston, MA USA

**Keywords:** Myeloma, CD38, Monoclonal antibody, Therapy

## Abstract

CD38 is a transmembrane glycoprotein that functions both as a receptor and an ectoenzyme, playing key roles in the regulation of calcium signaling and migration of immune cells to tumor microenvironments. High expression on multiple myeloma (MM) cells and limited expression on normal cells makes CD38 an ideal target for the treatment of MM patients. Two monoclonal antibodies directed at CD38, isatuximab and daratumumab, are available for use in patients with relapsed and/or refractory MM (RRMM); daratumumab is also approved in newly diagnosed MM and light-chain amyloidosis. Clinical experience has shown that anti-CD38 antibody therapy is transforming treatment of MM owing to its anti-myeloma efficacy and manageable safety profile. Isatuximab and daratumumab possess similarities and differences in their mechanisms of action, likely imparted by their binding to distinct, non-overlapping epitopes on the CD38 molecule. In this review, we present the mechanistic properties of these two antibodies and outline available evidence on their abilities to induce adaptive immune responses and modulate the bone marrow niche in MM. Further, we discuss differences in regulatory labeling between these two agents and analyze recent key clinical trial results, including evidence in patients with underlying renal impairment and other poor prognostic factors. Finally, we describe the limited existing evidence for the use of isatuximab or daratumumab after disease progression on prior anti-CD38 mono- or combination therapy, highlighting the need for additional clinical evaluations to define optimal anti-CD38 antibody therapy selection and sequencing in RRMM.

## Introduction

CD38 is a 45-kDa type-II transmembrane glycoprotein that serves as both an important receptor and ectoenzyme [1, 2]. The receptor roles of its large extracellular domain rely on frontal and lateral interactions with other functional receptors to modulate various immune functions [3–5]. Through interaction with CD31, its main ligand CD38 [6] induces leukocyte activation, proliferation, and migration through the endothelial cell wall and differentiation of B cells [2–4]. The ectoenzymatic activity of CD38 is independent of its receptor functions [1] and leads to synthesis of cADPR and NAADP from NAD and NADP, respectively, thereby producing key secondary messengers that mobilize calcium from intracellular stores and regulate calcium signaling [4, 7–9]. CD38 enzymatic activity also leads to extracellular adenosine production, which is immunosuppressive and may contribute to immune system evasion by tumor cells [5, 10].

CD38 expression is absent on early hematopoietic progenitors, but variable and generally low on normal myeloid and lymphoid cells. Expression is highest on plasma cells and multiple myeloma (MM) cells, making it an ideal target for MM therapy. Importantly, CD38 is also expressed on cells of the innate immune system, including natural killer (NK) cells, and on cells outside of immunologic networks such as red blood cells (RBCs) and platelets [1, 4–6, 11].

Two anti-CD38 monoclonal antibodies, isatuximab (humanized immunoglobulin [Ig]G1,κ) and daratumumab (human IgG1,κ), are currently approved for MM patients [5, 12–16]. Isatuximab is approved in various countries in combination with pomalidomide and dexamethasone (Pd) for RRMM patients (≥ 2 prior therapy lines), based on the ICARIA-MM study. To date, isatuximab is also approved in combination with carfilzomib and dexamethasone (Kd) for patients with relapsed MM (1–3 prior lines) in the USA and for MM patients who have received ≥ 1 prior therapy in the European Union, based on the IKEMA study [12, 15, 16]. Daratumumab is approved as monotherapy and in combination with IMiD drugs (i.e., lenalidomide or pomalidomide based on the POLLUX, EQUULEUS, and APOLLO trials) or PIs (i.e., bortezomib or carfilzomib based on the CASTOR and CANDOR trials), plus dexamethasone for RRMM patients. Daratumumab is also approved in combination with bortezomib-melphalan-prednisone, bortezomib-thalidomide-dexamethasone, or lenalidomide-dexamethasone in newly diagnosed MM (NDMM), based on the ALCYONE, CASSIOPEIA, and MAIA trials, respectively, and in combination with bortezomib-cyclophosphamide-dexamethasone for light-chain amyloidosis, based on the ANDROMEDA trial [13, 14, 17, 18]. Anti-CD38 antibodies are transforming MM treatment owing to their profound anti-myeloma activity as single agents and in combinations, as well as their manageable safety profiles [5].

In this review, we will present available evidence on the similarities and differences existing between isatuximab and daratumumab, ranging from their mechanisms of action to translational results, and an overview of recent, key clinical study findings for each agent, to better define their role in the management of patients with RRMM.

## Mechanisms of action

**Fig. 1:**
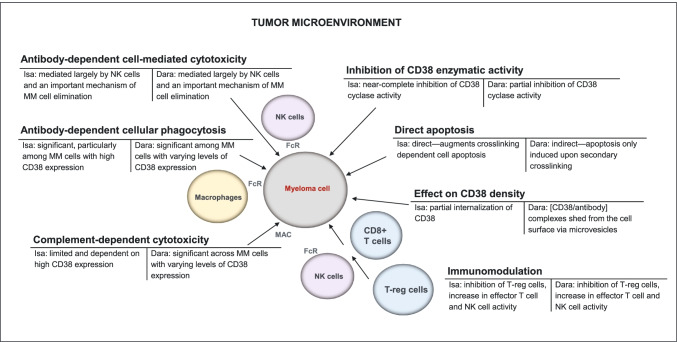
Mechanisms of action of the anti-CD38 monoclonal antibodies isatuximab and daratumumab*. Dara*, daratumumab; *FcR*, Fc receptor; *Isa*, isatuximab; *MAC*, membrane attack complex; *MM*, multiple myeloma; *NK*, natural killer; *T-reg*, regulatory T cell

Anti-CD38 antibodies exert their therapeutic effects via direct effector mechanisms on MM cells (i.e., CD38 enzymatic inhibition, direct induction of apoptosis) and through Fc-dependent immune mechanisms, including antibody-dependent cellular cytotoxicity (ADCC), complement-directed cytotoxicity (CDC), and antibody-directed cellular phagocytosis (ADCP) (Fig. 1 for a summary of mechanisms of action known for isatuximab and daratumumab).

### CD38 binding and enzymatic inhibition

Mechanistic differences between isatuximab and daratumumab likely stem from their binding to different, non-overlapping epitopes on the L-shaped CD38 molecule [3, 4, 19]. The CD38 active site is located in a pocket near the center of the molecule [7]. Isatuximab binds to a specific epitope that partially encompasses, but does not block access to or alter the configuration of the CD38 ectoenzyme catalytic site [11, 20]. In contrast, the daratumumab binding site is located completely outside the CD38 catalytic site [11].

The unique binding sites of isatuximab and daratumumab may explain their differential in vitro abilities to inhibit the CD38 enzymatic activity and to induce different structural changes within the CD38 molecule upon binding. Exposure of recombinant CD38 + cells to isatuximab produced near-complete inhibition of the CD38 cyclase activity in a dose-dependent manner, while both a surrogate antibody for daratumumab (produced based on published sequences and thus potentially not completely identical to the original antibody) and daratumumab resulted in a much lower inhibition [19, 21, 22]. Partial inhibition by daratumumab could not be overcome by increasing antibody concentrations, as evidenced in additional experiments with both recombinant CD38 protein and CD38-expressing cells [23].

### CD38 expression

The ability of anti-CD38 antibodies to induce cell death is at least partially dependent on CD38 expression on the MM cell surface. Thus, maintenance of CD38 expression is likely important for continued responsiveness of MM cells to anti-CD38 therapy. Continuous exposure of various MM cell lines to effective concentrations of isatuximab induces internalization of CD38, but not its significant release from the surface of MM cells [24]. On the other hand, CD38 ligation by daratumumab leads to aggregation of protein:antibody complexes, formation of distinct polar aggregates, and subsequent release of microvesicles (MVs). Prior to eventual internalization by distant immune cells, the formation of MVs, which also contain important immune-cell receptors and inhibitory complement receptors, may contribute to an immune or tolerogenic microenvironment for MM cells in the bone marrow (BM) niche [25] [26].

### Induction of apoptosis

Both isatuximab and daratumumab can induce apoptosis by crosslinking with Fcγ receptors on immune effector cells [27–29]. Isatuximab was selected for clinical development based on its additional ability to induce direct cell death, as demonstrated in both in vitro and ex vivo studies [21]. When compared with a large panel of murine monoclonal antibodies that specifically bound to human CD38, isatuximab was the only antibody with potent apoptotic activity in the absence of crosslinking agents [21]. This ability was confirmed in studies using cells from BM aspirates of MM patients [21]. The direct MM cell-killing effect of isatuximab has since been shown to depend on both caspase-dependent and, to a greater extent, lysosomal cell-death pathways [29]. Recent studies confirmed that daratumumab lacks the ability to induce apoptosis in the absence of crosslinking agents, though the contribution of direct cell death to overall anti-CD38 killing of MM cells in patients remains to be fully characterized [28].

### ADCC

Both isatuximab and daratumumab induce similar levels of ADCC across MM cells with a broad range of CD38 expression [22, 24, 28]. Early preclinical studies showed that daratumumab effectively induced ADCC in a dose-dependent manner [30] and recent studies with isatuximab suggest that ADCC may be a more dominant effector mechanism than originally thought [24, 31]. ADCC is largely mediated by binding of the IgG-Fc region to Fcγ receptors on the surface of NK cells [32]. In addition to triggering ADCC, CD38 ligation can induce direct activation of NK cell cytotoxicity and, although it may lead to depletion of CD38^high^ NK cells, it retains the population of cytotoxic NK cells [32, 33]. Indeed, recent studies showed that, in addition to mediating ADCC in in vitro and ex vivo models, isatuximab and daratumumab directly activated NK cells and increased their lytic activity against tumor cells through CD16 and CD38 crosslinking [32, 33].

### CDC

Daratumumab was selected among a panel of other anti-CD38 monoclonal antibodies based on its ability to induce CDC in early in vitro studies with CD38^+^ cell lines [30]. In ex vivo experiments, daratumumab also induced concentration-dependent, complement-mediated lysis of MM cells freshly isolated from patients [30]. Further studies showed that CDC appears to be a more prominent effector mechanism for daratumumab [22, 28], with evidence suggesting that isatuximab induces CDC only in the presence of high CD38 receptor density [24, 32]. MM patients who progressed after daratumumab treatment had elevated levels of the complement inhibitors CD55 and CD59, in agreement with the hypothesis that CDC represents a major mechanism of action for daratumumab and suggesting a mechanism of resistance to CDC-mediating antibodies. Consistently, blocking CD55 and in particular CD59 was found to increase in vitro CDC of MM cells induced by daratumumab [34, 35].

### ADCP

ADCP is a rapid and potent mechanism of action for daratumumab across MM cells of differing CD38 expression [36]. A significant expansion of M1 inflammatory macrophages and depletion of plasmacytoid dendritic cells (DCs) were reported in patients sensitive to treatment with daratumumab-Pd, using a single-cell sequencing approach [37]. Isatuximab was also shown to induce significant ADCP against cells with elevated CD38 expression [24]. Recent studies suggest that daratumumab and isatuximab induce similar levels of ADCP [28] and that ADCP is a primary mechanism for isatuximab-mediated cytotoxicity [32].

### “In-vivo vaccination” effect

The binding of antibodies to exogenous antigens (e.g., viral proteins) or tumor-associated antigens can promote their uptake, processing, and cross-presentation by antigen-presenting cells (e.g., DCs), ultimately leading to priming of CD8^+^ T cell effectors and induction of memory responses [38, 39]. This pathway is thought to be important for mounting both cell-mediated and humoral adaptive immune responses against specific tumor antigens and viral infections [38, 39].

Atanackovic et al. described an “in-vivo vaccination” response to isatuximab in 4 RRMM patients [39]. Following exposure to isatuximab, patients with tumor-specific immune “fitness” at baseline developed additional antibody responses to myeloma-associated antigens including MAGE-C2 and NY-ESO-1 [39]. These patients also demonstrated increasing T cell responses against CD38 and clinical myeloma responses to treatment [39].

Malavasi et al. recently hypothesized that sequestration of daratumumab:CD38 complexes into MVs may ultimately facilitate an “autovaccination” effect [26]. Downstream internalization of MVs by immune cells, such as DCs, may activate immune responses against MM cells and lead to an improved overall immunological response [26].

### Modulation of the immunosuppressive tumor microenvironment

The BM niche in MM patients is intrinsically immunosuppressive, containing cells able to blunt the immune response and adenosine (ADO) that may lead to an anergic status of T, NK, and dendritic cells [10]. High ADO levels in the tumor microenvironment may correlate with disease progression and represent a mechanism for downregulation of T cell effector functions [40, 41]. The CD38 hydrolase activity is largely responsible for the conversion of NAD^+^ to ADO within the hypoxic, acidic MM niche [25]. Although hypothetical, differential CD38 hydrolase inhibition by anti-CD38 antibodies might result in divergent abilities to suppress the formation of ADO. The inhibitory or enhancing effects of isatuximab and daratumumab on the hydrolase activity of CD38 molecules in MM cells [19, 21, 23, 40] and the potential in vivo impact on ADO production in MM patients remain to be further investigated and clarified [11].

Within the tumor microenvironment, immune and nonimmune cells cooperate to promote tumor survival and immune system evasion [42]. In this setting, CD38 is expressed on several immunosuppressive cell types including regulatory T cells (Tregs), regulatory B cells (Bregs), myeloid-derived suppressor cells (MDSCs), and NK cell subsets [42]. Targeting these CD38-expressing cells may help restore immune responses against tumor cells. Indeed, anti-CD38 antibodies demonstrated the ability to modulate the immunosuppressive microenvironment within the context of MM [43]. In ex vivo studies, Feng et al. showed that isatuximab can preferentially block Tregs and enhance NK and CD8^+^ T-effector cell-mediated immune responses, effectively restoring immune effector functions against MM cells [31]. This ability was enhanced by IMiD drugs, more potently by pomalidomide than lenalidomide [31]. Similarly, Krejcik et al. showed that daratumumab can reduce CD38^+^ Treg, Breg, and MDSC cell populations in ex vivo studies, while inducing helper and cytotoxic T cell expansion [44]. The ability to increase T cell clonality in RRMM patients was reported for both anti-CD38 agents in clinical trials [44, 45]. Future studies may help to determine the role of immunomodulatory effects within the greater context of clinical responses to isatuximab and daratumumab.

## Translational medicine

### Interference with indirect Coombs tests

As CD38 is widely expressed on RBCs, both isatuximab and daratumumab can interfere with indirect Coombs tests used for blood compatibility testing [46, 47]. This interference may complicate the safe release of blood products for MM patients who are frequently anemic and may require blood transfusion. The phenomenon was first recognized during phase I/II studies, when the plasma of all daratumumab-treated MM patients demonstrated false-positive antibody screens and pan-reactivity on RBC-panel testing for up to 6 months after treatment discontinuation [46, 47]. Thus, prior to initiating daratumumab, patients should have extended RBC phenotyping/genotyping and transfusion laboratories must be notified [13]. Isatuximab shares this requirement, as clinical trial results demonstrated interference with indirect Coombs tests, although to a lower extent (63–68% of patients) [15, 48].

This difference may be explained by the results of in vitro binding studies, which showed that daratumumab can directly bind CD38 on RBCs, whereas isatuximab requires a cofactor (e.g., a mouse anti-CD38 antibody or a CD38 inhibitor), suggesting masking of the isatuximab epitope on RBCs in some patients [49, 50].

Several strategies for overcoming interference have been explored, including RBC treatment with dithiothreitol (DTT) or trypsin [49, 50]. DTT, which denatures CD38 and prevents antibody binding, represents the most accepted strategy for mitigating interference [50]. However, DTT denatures other significant RBC antigens, most notably the Kell antigen [50, 51]. Thus, all patients need to be transfused with Kell-compatible blood, unless Kell positivity was established prior to anti-CD38 treatment [50]. For transfusion laboratories that cannot perform DTT- or trypsin-treatment techniques, transfusion of ABO Rh(D)- and extended RBC phenotype/genotype-compatible blood are advised for patients receiving either anti-CD38 antibody [51].

Mitigating blood-typing interference by anti-CD38 antibodies can be time-consuming and expensive. Recently, Izaguirre et al. established a modified DTT technique that requires lower DTT concentrations, is less time-consuming, and appears to avoid denaturation of Kell antigen [52].

### Immunofixation interference

Furthermore, administered human IgG1,κ monoclonal antibodies can interfere with the detection of clonal IgG,κ molecules in the sera of MM patients, as measured by serum protein electrophoresis and/or immunofixation electrophoresis (IFE), owing to comigration in the assays [53]. Such interference is of importance as it may lead to underestimate clinical response to treatment in some cases, particularly in the determination of complete response (CR) [48, 53, 54]. As reported for isatuximab and daratumumab, mass spectrometry analyses and the use of specific IFE-reflex assays (i.e., isatuximab Hydrashift assay, DIRA assay) can overcome interference by these therapeutic antibodies and allow more accurate measurements of serum M-protein in MM patients [48, 53–55]. As indicated in a recent report of the International Myeloma Working Group Mass Spectrometry Committee, although requiring assessment in a specialized laboratory, mass spectrometry can be effectively used to evaluate M-protein levels in treated patients and achieve high sensitivity in detecting residual serum M-protein. In this method, M-protein can be assessed by immuno-capture and liquid chromatography coupled to high-resolution mass spectrometry, without potential interference by a therapeutic antibody. However, its high sensitivity in detecting minimal residual disease (MRD) may lead to lower CR response rates, thus hindering comparisons with results obtained by immunofixation at different time points or in other studies [56].

## Trial design and regulatory labeling

### Trial design in pomalidomide and carfilzomib combinations for RRMM and regulatory labeling

In the pivotal trials that led to their approvals, both isatuximab and daratumumab were evaluated in combination regimens with pomalidomide or carfilzomib in RRMM patients, who had received ≥ 1 prior line of anti-myeloma treatment, including older patients (aged > 65–70 years) and patients with decreased renal function or cytogenetic abnormalities [12–18].

In combination with Pd or Kd, isatuximab (10 mg/kg) is administered by IV infusion on days 1, 8, 15, and 22 (weekly, QW) in cycle 1 and on days 1 and 15 (every 2 weeks, Q2W) from cycle 2. Daratumumab is administered by IV infusion in combination with Pd at 16 mg/kg QW for 8 weeks, Q2W in weeks 9–24, and Q4W from week 25, and in combination with Kd at 8 mg/kg on days 1 and 2 in week 1, followed by 16 mg/kg QW in weeks 2–8, Q2W in weeks 9–24, and Q4W from week 25 [13, 15].

A subcutaneous (SC) formulation of daratumumab is approved for use and can be administered over ~ 5 min (QW for 8 weeks, Q2W in weeks 9–24, and Q4W from week 25, as monotherapy or combined with Pd or lenalidomide-dexamethasone) [14]. Non-inferiority of SC vs. IV daratumumab was demonstrated in RRMM patients for monotherapy in the Phase III COLUMBA trial (≥ 3 prior lines) and in standard combination regimens in the Phase II PLEIADES trial (≥ 1 prior lines) [14]. SC administration of daratumumab decreases the risk for infusion reactions (IRs) to ~ 15%, but carries the same requirements for pre- and post-administration corticosteroid prophylaxis as the IV formulation [14]. A SC version of isatuximab is currently under investigation in patients with RRMM (NCT04045795) [57].

Both monoclonal antibodies can cause IRs following IV administration, generally of grade 1–2 and within the first treatment cycle (mainly after the first dose). To prevent IRs, both isatuximab and daratumumab require systemic premedication with corticosteroids, acetaminophen, and antihistamines [13, 15]. Although incidence and severity of IRs may depend on the infusion speed, a shorter duration of IV administration for isatuximab is not associated with increased IRs nor it requires post-infusion steroid prophylaxis [15, 58].

A difference exists between the isatuximab and daratumumab trial designs for patients with chronic obstructive pulmonary disease (COPD) or other respiratory diseases, which are comorbidities that may affect outcomes in MM [59]. In ICARIA-MM (NCT02990338, phase III trial, isatuximab-Pd), there were no pulmonary exclusions; 10% of patients had concomitant COPD or asthma at baseline [48]. In EQUULEUS, COPD patients with FEV_1_ < 50% and patients with moderate/severe or uncontrolled asthma were excluded [60]. APOLLO (NCT03180736, phase III trial, daratumumab-Pd) excluded patients with COPD and FEV_1_ < 50% [61]. Similar discrepancies exist between phase III trials of isatuximab and daratumumab in combination with Kd [62, 63]. In IKEMA (NCT03275285, isatuximab-Kd), there were no pulmonary exclusions, whereas in CANDOR (NCT03158688, daratumumab-Kd), patients with known COPD and FEV_1_ < 50% were excluded, as were those with known moderate/severe persistent asthma [62–64].

The exclusion of patients with COPD and asthma in key daratumumab studies and the incidence of bronchial hyperreactivity AEs seen in early trials resulted in the recommendation for post-administration prophylaxis with bronchodilators and inhaled corticosteroids in patients with a history of COPD [13, 65]. Prophylaxis may be discontinued after the first 4 daratumumab doses, if the patient has not experienced a major IR [13]. Bronchodilator prophylaxis is not required after isatuximab administration [15].

## Clinical trial results

### Pomalidomide and carfilzomib combinations

Both isatuximab and daratumumab were evaluated in combination with Pd and Kd in randomized phase III trials. Results highlighted in this review are from different, independent trials.

#### ICARIA-MM and APOLLO

In preclinical studies, pomalidomide was found to augment the direct apoptotic and immunomodulatory activities of isatuximab [29, 31]. Following encouraging results in a phase Ib dose-escalation study [66], the addition of isatuximab to Pd was further assessed in the Phase III ICARIA-MM trial [48]. Results of ICARIA-MM led to the first approval for isatuximab use in patients with RRMM [15].

ICARIA-MM was a randomized, multicenter, open-label study conducted in 24 countries across North America, Europe, and the Asia–Pacific region [48]. RRMM patients (*N* = 307) who had received ≥ 2 prior treatment lines (including lenalidomide and a PI) were randomized to receive isatuximab-Pd (*n* = 154) or Pd alone (*n* = 153). The primary endpoint was progression-free survival (PFS). At median follow-up of 11.6 months, patients in the isatuximab-Pd arm achieved a median PFS of 11.5 months vs. 6.5 months in the Pd arm (HR, 0.596; 95% CI, 0.44–0.81; *P* = 0.001) [48]. Key patient characteristics and outcomes are presented in Table 1.

**Table 1:** Results of isatuximab and daratumumab randomized phase 3 trials in combination with pomalidomide and dexamethasone in patients with RRMM

		ICARIA-MM ^48^ Isa-Pd *N* = 307		APOLLO ^61,68^ Dara-Pd *N* = 304	
Administration		IV		IV and SC^a^	
Median lines of prior therapy, n		3		2	
Refractory to Len/PI/Len + PI, %		93/76/71		80/48/42	
High-risk cytogenetics^b^, %		20		35^c^	
Regimen		Isa-Pd (*n* = 154)	Pd (*n* = 153)	Dara-Pd (*n* = 151)	Pd (*n* = 153)
Median follow-up, mo		11.6		16.9	
Efficacy	Median PFS, mo	11.5	6.5	12.4	6.9
	PFS HR (95% CI) *P*-value	0.596 (0.44–0.81) *P* = .001		0.63 (0.47–0.85) *P* = .0018	
	ORR, %	60.4	35.3	69	46
	≥ VGPR, %	31.8	8.5	51	19.6
	≥ CR^d^, %	4.5	2	24.5	3.9
	MRD-, %	5	0	9	2
Safety	Neutropenia (≥ grade 3), %	85	70	68	51
	Discontinued due to AE, %	7.2	12.8	2	3

*AE*, adverse event; *CI*, confidence interval; *CR*, complete response; *d*, dexamethasone; *Dara*, daratumumab; *HR*, hazard ratio; *Isa*, isatuximab; *IV*, intravenous; *mo*, months; *MRD*, minimal residual disease; *ORR*, overall response rate; *P*, pomalidomide; *PFS*, progression-free survival; *SC*, subcutaneous; *VGPR*, very good partial response

^a^ Prior to protocol amendment, patients received daratumumab IV; after protocol amendment, all patients received daratumumab SC co-formulated with recombinant human hyaluronidase

^b^ Based on fluorescence in situ hybridization; defined as presence of del(17p), t(14;16), or t(4;14). ^c^ Reported in assessable patients. ^d^ Rates reported reflect patients achieving either complete response or stringent complete response

APOLLO was a randomized, multicenter, open-label study conducted across 12 countries [61, 67]. RRMM patients (*N* = 304) who had received ≥ 1 prior therapy line (including lenalidomide and a PI) were randomized to receive daratumumab-Pd (*n* = 151) or Pd alone (*n* = 153). Similar to ICARIA-MM, the primary endpoint was PFS. At median follow-up of 16.9 months, patients in the daratumumab-Pd arm achieved a median PFS of 12.4 months vs. 6.9 months in the Pd arm (HR, 0.63; 95% CI, 0.47–0.85; *P* = 0.0018) (Table 1) [61].

There were several differences in patient populations between the ICARIA-MM and APOLLO trials (Table 1). Overall, ICARIA-MM enrolled a more refractory patient population compared with APOLLO: at baseline, patients in ICARIA-MM had received more prior therapy lines (median, 3 vs. 2 lines in APOLLO) and were more refractory to lenalidomide (93 vs. 80%), a PI (76 vs. 48%), or both (71 vs. 42%) [48, 61, 68]. However, patients in APOLLO were more likely to have high-risk cytogenetics, defined as presence of del(17p), t(14;16), or t(4;14), although cutoff levels were not reported. Cutoffs for high-risk cytogenetics in ICARIA-MM were 50% for del(17p), and 30% for t(4;14) and t(14;16), as assessed by fluorescence in situ hybridization during central laboratory screening [48].

Response rates are listed for both trials in Table 1. Five percent of patients reached MRD negativity with isatuximab-Pd vs. 0% with Pd in ICARIA and 9% of patients with daratumumab-Pd vs. 2% with Pd in APOLLO [12, 61, 68]. MRD negativity was assessed in ICARIA-MM only for patients achieving CR/sCR and rates may have been underestimated due to interference of isatuximab with the assessment of M-protein levels via immunofixation [48]. In ICARIA-MM, grade ≥ 3 neutropenia was reported in 85% of patients in the isatuximab-Pd arm vs. 70% in the Pd arm and the discontinuation rate due to AEs was 7.2 vs. 12.8% with Pd, reflecting the extent of prior treatment in these patients. In APOLLO, grade ≥ 3 neutropenia was reported in 68% of patients in the daratumumab-Pd arm vs. 51% in the Pd arm, with a discontinuation rate of 2 vs. 3% with Pd [48, 61, 68].

In both ICARIA-MM and APOLLO, the respective anti-CD38 antibodies yielded PFS benefit across almost all subgroups studied [48, 61, 68]. Data for selected subgroups are presented in Table 2. Additional subgroup analyses were performed for ICARIA-MM patients with soft-tissue plasmacytomas and gain(1q21) at baseline, both of which are associated with poorer prognosis in MM patients [69, 70]. Treatment with isatuximab-Pd significantly improved median PFS in patients with plasmacytomas (HR, 0.22; 95% CI, 0.07–0.69) and in patients with isolated gain(1q21) (HR, 0.50; 95% CI, 0.28–0.88) compared with Pd [69, 70]. Analysis of patients with baseline renal impairment (estimated glomerular filtration rate [eGFR] < 60 mL/min/1.73 m^2^) was also conducted for ICARIA-MM [71]. Complete renal response (eGFR improvement from < 50 at baseline to ≥ 60 mL/min/1.73 m^2^ in ≥ 1 post-baseline assessment) was achieved in 72% of patients on isatuximab-Pd vs. 38% on Pd. Renal response was more durable (lasting ≥ 60 days) with isatuximab-Pd and tumor response rates were higher in patients on isatuximab-Pd who achieved a renal response (52 vs. 44% on Pd) [71].

**Table 2:** Selected subgroup analyses from isatuximab and daratumumab randomized phase 3 trials in combination with pomalidomide and dexamethasone

Subgroup	ICARIA-MM ^48,69,70^ Isa-Pd vs. Pd		APOLLO ^61,68^ Dara-Pd vs. Pd	
	PFS HR (95% CI)	P_interaction_	PFS HR (95% CI)	P_interaction_
Age		0.5952		NR
< 65 yr	0.66 (0.40–1.07)		0.69 (0.44–1.09)	
≥ 65 yr	65–74 yr: 0.64 (0.39–1.06) ≥ 75 yr: 0.48 (0.24–0.95)		0.55 (0.38–0.81)	
Previous lines of therapy		0.9583		NR
1	–^a^		0.70 (0.30–1.67)	
2–3	0.59 (0.40–0.88)		0.66 (0.48–0.92)	
> 3	0.59 (0.36–0.98)		0.40 (0.18–0.90)	
Baseline renal function^b^	eGFR (mL/min/1.73 m^2^) ≥ 60: 0.58 (0.38–0.88) < 60: 0.50 (0.30–0.85)	0.7575	CrCl (mL/min) > 60: 0.64 (0.45–0.90) ≤ 60: 0.59 (0.35–0.99)	NR
Cytogenetic risk^c^		0.9990		NR
High risk	0.66 (0.33–1.28)		0.85 (0.49–1.44)	
Standard risk	0.62 (0.42–0.93)		0.51 (0.32–0.81)	
Soft-tissue plasmacytomas	0.22 (0.07–0.69)	NR	NA	
Gain 1q21	0.50 (0.28–0.88)	NR	NA	

*CI*, confidence interval; *CrCl*, creatinine clearance; *d*, dexamethasone; *Dara*, daratumumab; *eGFR*, estimated glomerular filtration rate; *HR*, hazard ratio; *Isa*, isatuximab; *MDRD*, Modification of Diet in Renal Disease; *NA*, not analyzed; *NR*, not reported; *P*, pomalidomide; *PFS*, progression-free survival; *yr*, year

The differences in patient characteristics between the studies and limited sample size in the subgroup

Analyses prevent comparisons across trials

^a^All patients in ICARIA-MM had received at least 2 lines of prior therapy

^b^Measured as estimated glomerular filtration rate (by MDRD equation) in ICARIA-MM and as creatinine clearance (method not specified) in APOLLO

^c^Based on fluorescence in situ hybridization; high risk defined as del(17p), t(4;14), or t(4;16)

#### IKEMA and CANDOR

Results from a phase Ib study (NCT02332850) indicated that isatuximab combined with carfilzomib was clinically active in heavily pretreated RRMM patients [72]. The combination of isatuximab-Kd was further investigated in the Phase III IKEMA trial, a randomized, open-label study conducted across 16 countries in North America, South America, Europe, and the Asia–Pacific region [54, 64].

IKEMA enrolled patients (*N* = 302) with relapsed MM who had received 1–3 prior therapy lines. Patients were randomized 3:2 to isatuximab-Kd (*n* = 179) or Kd alone (*n* = 123). The primary endpoint was PFS. At a median follow-up of 20.7 months, the addition of isatuximab to Kd yielded a statistically significant improvement in PFS (HR, 0.531; 99% CI 0.318–0.889; one-sided *p* = 0.0007) [54]. Cytogenetic risk and MRD were assessed by central laboratory. The MRD negativity (MRD-) rate was 29.6% with isatuximab-Kd vs. 13.0% with Kd (*p* = 0.0004) [54, 64]. Key patient characteristics and outcomes are presented in Table 3. Consistent with the interim analysis, results of the updated PFS analysis at a median follow-up of 44 months showed a median PFS of 35.7 (95% CI 25.8–44.0) months with isatuximab-Kd vs. 19.2 (95% CI 15.8–25.0) months with Kd (HR, 0.58; 95.4% CI 0.42–0.79) [73].

**Table 3:** Results of isatuximab and daratumumab randomized phase 3 trials in combination with carfilzomib and dexamethasone in patients with relapsed MM

		IKEMA ^54,64^ Isa-Kd *N* = 302		CANDOR ^75^ Dara-Kd *N* = 466	
Administration		IV		IV	
Median lines of prior therapy, n		2		2	
Refractory to Len/PI, %		32.8/33.1		33/29	
High-risk cytogenetics^a^,%		24.2		16	
Regimen		Isa-Kd (*n* = 179)	Kd (*n* = 123)	Dara-Kd (*n* = 312)	Kd (*n* = 154)
Median follow-up, mo		20.7	20.9	16.9	16.3
Efficacy	Median PFS, mo	NR	19.2	NR	15.8
	PFS HR (CI) *P*-value	0.53 (99% CI, 0.32–0.89) *P* = .0007		0.63 (95% CI, 0.46–0.85) *P* = .0027	
	ORR, %	86.6	82.9	84	75
	≥ VGPR, %	72.6	56.1	69	49
	CR, %	39.7^b^	27.6	29	10
	MRD-^c^, %	29.6	13.0	18	4
Safety	TEAE (≥ grade 3), %	76.8	67.2	82	74
	Discontinued due to AE, %	8.5	13.9	22	25

*AE*, adverse event; *CI*, confidence interval; *CR*, complete response; *d*, dexamethasone; *Dara*, daratumumab; *HR*, hazard ratio; *Isa*, isatuximab; *IV*, intravenous; *K*, carfilzomib; *mo*, months; *MRD*, minimal residual disease; *NR*, not reached; *ORR*, overall response rate; *PFS*, progression-free survival; *SC*, subcutaneous; *TEAE*, treatment-emergent adverse event; *VGPR*, very good partial response

^a^ Based on fluorescence in situ hybridization; high risk defined as del(17p), t(4;14), or t(4;16)

^b^ Represents an underestimate of complete response due to interference of isatuximab with immunofixation techniques used to measure M-protein levels. Potential adjusted CR rate was 45.8%

^c^ MRD negativity was assessed at any timepoint after the first dose of study treatment in ≥ VGPR patients in IKEMA and at the 12-month landmark (from 8 to 13 months window) in all patients in CANDOR

Based on the encouraging clinical response seen with daratumumab-Kd in EQUULEUS [74], a phase III study (CANDOR) was conducted to further evaluate daratumumab-Kd in RRMM patients who had received 1–3 prior therapy lines [75]. Patients (*N* = 466) were randomized 2:1 to either daratumumab-Kd (*n* = 312) or Kd alone (*n* = 154). Similar to IKEMA, the primary outcome in CANDOR was PFS. After a median follow-up of 16.9 months, the addition of daratumumab to Kd yielded a statistically significant improvement in median PFS (HR, 0.63; 95% CI 0.46–0.85; two-sided *P* = 0.0027) (Table 3) [75]. Results from the updated PFS analysis at a median follow-up of 27.8 months in the daratumumab-Kd group and 27.0 months in the Kd group showed a median PFS of 28.6 months (95% CI 22.7–not estimable) with daratumumab-Kd vs. 15.2 months (95% CI 11.1–19.9) with Kd (HR, 0.59, 95% CI 0.45–0.78, log-rank *p* < 0.0001) [76].

Overall, patient populations studied in IKEMA and CANDOR were quite similar, with a median of 2 prior treatment lines and similar refractoriness to lenalidomide (33 vs. 33%) and a PI (33 vs. 29%) [54, 64, 75]. Patients in IKEMA appeared more likely to have high-risk cytogenetics, though a higher percentage of patients in CANDOR had unknown cytogenetics at baseline compared with IKEMA (51 vs. 12%) [54, 75]. CR rates were underestimated in IKEMA due to interference of isatuximab with the serum immunofixation test required for CR; the adjusted CR rate was estimated to be 45.8% rather than 39.7% [55]. MRD was assessed in ≥ VGPR patients in the two studies, when best response was reached in IKEMA and at fixed time points in CANDOR. MRD- rate in the intent-to-treat population was 29.6% in IKEMA and 17.6% in CANDOR at 12 months. The incidence of treatment-emergent AEs was similar between trials. The rate of discontinuation due to AEs was 8.5% with isatuximab-Kd vs. 13.9% with Kd in IKEMA and 22% with daratumumab-Kd vs. 25% with Kd in CANDOR [54, 64, 75].

In both IKEMA and CANDOR, the respective anti-CD38 antibody yielded PFS benefit across almost all subgroups studied [54, 64, 75]. Data for selected subgroups are presented in Table 4. Complete renal response (reversal of renal impairment) was assessed in IKEMA and occurred in 52% of patients in the isatuximab-Kd arm vs. 31% in the Kd arm. Renal response was also more likely to be durable (lasting ≥ 6 months) in the isatuximab-Kd group [54, 64]. Complete renal response was not assessed for patients in CANDOR, although PFS benefit was observed across all ranges of baseline renal function studied [75].

**Table 4:** Selected subgroup analyses from isatuximab and daratumumab randomized phase 3 trials in combination with carfilzomib and dexamethasone

Subgroup	IKEMA ^54,64^ Isa-Kd vs. Kd		CANDOR ^75^ Dara-Kd vs. Kd	
	PFS HR (95% CI)	P_interaction_	PFS HR (95% CI)	P_interaction_
Age	< 65 yr: 0.64 (0.37–1.11) ≥ 65 yr: 0.43 (0.25–0.74)	0.3663	≤ 65 yr: 0.57 (0.38–0.86) > 65 yr: 0.76 (0.48–1.22)	0.3653
Previous lines of therapy		0.6841		0.7230
1	0.59 (0.31–1.12)		0.68 (0.40–1.14)	
≥ 2	0.48 (0.29–0.78)		0.61 (0.42–0.88)	
Baseline renal function^a^	eGFR (mL/min/1.73 m^2^) ≥ 60: 0.63 (0.39–1.00) < 60: 0.27 (0.11–0.66)	0.1101	CrCl (mL/min) ≥ 80: 0.68 (0.44–1.03) ≥ 50 to < 80: 0.65 (0.36–1.15) ≥ 15 to < 50: 0.44 (0.19–1.00)	0.6445
Cytogenetic risk^b^		0.2707		0.6887
High risk	0.72 (0.36–1.45)		0.70 (0.36–1.40)	
Standard risk	0.44 (0.27–0.73)		0.50 (0.28–0.90)	
Gain 1q21	0.57 (0.33–0.98)	0.5572	NR	

*CI*, confidence interval; *CrCl*, creatinine clearance; *d*, dexamethasone; *Dara*, daratumumab; *eGFR*, estimated glomerular filtration rate; *HR*, hazard ratio; *Isa*, isatuximab; *K*, carfilzomib; *MDRD*, modification of diet in renal disease; *NR*, not reported; *PFS*, progression-free survival; *yr*, year

The differences in patient characteristics between the studies and limited sample size in the subgroup analyses prevent comparisons across trials

^a^Measured as estimated glomerular filtration rate (by MDRD equation) in IKEMA and as creatinine clearance (method not specified) in CANDOR

^b^Based on fluorescence in situ hybridization; high risk defined as del(17p), t(4;14), or t(4;16)

### Anti-CD38 antibody sequencing

How anti-CD38 antibodies should be sequenced and whether additional anti-myeloma activity can be achieved from an anti-CD38 antibody in RRMM patients already exposed to anti-CD38 therapy are important clinical questions currently being addressed by investigators. Initial findings were recently reported from monotherapy and combination therapy trials.

#### Monotherapy

Results of studies with either single-agent isatuximab after daratumumab or daratumumab after isatuximab have provided evidence of limited efficacy for anti-CD38 antibody monotherapy in RRMM. A phase I/II study (NCT02514668) assessed the response to isatuximab monotherapy in daratumumab-refractory patients [77]. Enrolled patients (*N* = 32) were heavily pretreated, with a median of 7 prior lines (60% had received daratumumab combination therapy immediately prior to isatuximab initiation and 15% had received daratumumab in ≥ 2 prior lines). The overall disease control rate (defined as minimal response [MR] or better, or stable disease [SD] for ≥ 8 weeks) was 37.5% and, notably, was twofold higher in patients with a longer interval between the last daratumumab dose and isatuximab initiation (58.3% with ≥ 6-month washout after daratumumab vs. 28.6% with a < 3 months washout). An ORR was not reached, as only 1 patient achieved a MR (3.1%) and the others had SD (53.1%) or progressive disease [77].

It is known that reduction in CD38 expression on BM and circulating MM cells occurs rapidly after daratumumab initiation with significantly lower expression levels after the first infusion compared with baseline (potentially due to elimination of MM cells with high CD38 levels, endocytosis, or release in microvesicles), that may persist for at least 6 months after discontinuation [35]. Additionally, lower responses to anti-CD38 antibodies have been associated with low CD38 expression. For example, ADCC mediated by daratumumab against primary MM cells was 14.2% in cells within the lowest tertile of CD38 expression vs. 45.6% in cells of the highest tertile [78]. Taken together, these effects may explain the differences in disease control rates in this trial and offer insight as to the appropriate interval between different anti-CD38 antibody therapies.

Furthermore, updated results from the ICARIA-MM trial recently showed that daratumumab monotherapy appeared less effective after isatuximab-Pd than after Pd-only treatment. The ORR for daratumumab monotherapy or in combination with corticosteroids was 14.3% after isatuximab-Pd and 37.9% after Pd. In patients receiving daratumumab as first subsequent therapy, median PFS was lower in the isatuximab-Pd treated patients (2.2 months [95% CI 0.03–7.62], *n* = 9) than the Pd-treated patients (5.1 months [95% CI 3.75–10.51], *n* = 46) [79].

#### Combination therapy

Emerging efficacy results of combination therapy with either isatuximab after daratumumab or daratumumab after isatuximab may show a different trend compared with the monotherapy approach. In a phase Ib study (NCT02283775) of isatuximab-Pd administered by a fixed infusion volume, 7 of 47 patients had received daratumumab in a prior line [58]. One of these 7 patients achieved a partial response (PR), for an ORR of 14.3%; 2 (33.3%) patients had a MR and 3 (50.0%) had SD. Patients without prior daratumumab exposure achieved a 60% ORR (*n* = 40) [58].

To further characterize the efficacy of isatuximab after prior daratumumab exposure, investigators recently conducted a retrospective analysis of 15 patients treated with isatuximab-Pd after receiving daratumumab in prior lines [80]. Fourteen patients (89%) had previously had a PR to daratumumab; 13 patients discontinued daratumumab due to progressive disease and 2 due to toxicity. Despite prior daratumumab therapy, 8 patients (53%) achieved a PR and 2 patients (13%) a MR [80]. In addition, recent results from the ICARIA-MM trial showed that the ORR with daratumumab in combination with a PI, an IMiD, or an alkylating agent was similar in patients who had previously received isatuximab-Pd or Pd therapy (30.8 vs. 31.8%, respectively) [79].

Further experience with the use of anti-CD38 antibodies in RRMM after prior treatment with daratumumab will stem from a current phase Ib trial of SC isatuximab in combination with Pd (NCT04045795) and for daratumumab from a phase II study in combination with Kd (NCT03871829).

### Mechanisms of resistance

Patients with MM have shown benefit from anti-CD38 therapy in clinical trials. Nevertheless, they may eventually experience disease progression, indicating development of resistance to anti-CD38 antibody therapy. Several mechanisms of resistance have been proposed including downregulation of CD38 expression on MM cells, increased expression of complement inhibitory proteins, development of neutralizing antibodies, and depletion of NK cells [26, 81, 82].

In vitro and ex vivo studies have shown that CD38 expression levels correlate with isatuximab- and daratumumab-induced ADCC and CDC [78]. Additionally, CD38 expression on MM cells decreases rapidly during daratumumab treatment, is markedly reduced at time of progression, and may take up to 6 months to rebound after stopping daratumumab [35]. Proposed reasons for CD38 reduction with daratumumab include endocytosis, trogocytosis by granulocytes and monocytes, and packaging of daratumumab:CD38 complexes into MVs [82]. Corresponding data have not been reported for isatuximab.

Complement plays a key role in the killing effect of anti-CD38 antibodies [22, 28]. Importantly, daratumumab exerts greater CDC in the presence of low levels of complement inhibitors such as CD55 and CD59 [35]. Similarly, low expression levels of CD55 and CD59 on target cells favor isatuximab-mediated CDC [32]. Increased expression of both CD55 and CD59 has been seen on MM cells during disease progression on daratumumab treatment, indicating that overexpression of complement inhibitors is likely tied to daratumumab resistance [35].

Furthermore, emerging data on the effects of anti-CD38 antibodies on immune networks, suggest that immune-mediated mechanisms of resistance may occur following anti-CD38 treatment. In a recent report, MM cells from patients who discontinued treatment with daratumumab, due to disease progression, appeared to retain cell-surface expression of CD38 [33]. However, immune cells (e.g., CD8 + T cells and NK cells) showed impaired effector functions, with reduced or no killing of MM cells in an in vitro flow cytometry assay, compared with patients who responded to treatment [33]. Investigations in larger numbers of anti-CD38 antibody-treated patients may provide further evidence on the changes occurring among immune effectors within the tumor microenvironment of responders and non-responders, as well as insights on their relevance for selection of subsequent treatment.

## Conclusions

The anti-CD38 antibodies isatuximab and daratumumab represent important advances in the treatment of patients with RRMM. They display differences in their mechanisms of action, likely mediated by their binding to different, non-overlapping epitopes of the CD38 molecule. Undoubtedly, mechanisms of action are multifaceted and some mechanisms may predominate over others in different patients and in the context of different tumor microenvironments and immune milieu [34]. Further in vitro and ex vivo studies may contribute to our understanding of how mechanistic differences between these two antibodies might translate to better clinical outcomes in MM patients.

Phase III clinical trial results with isatuximab and daratumumab in RRMM have shown substantial improvements in PFS when used in combination with either Pd or Kd. Further detailed subgroup analyses were conducted with isatuximab in patients with renal impairment, soft-tissue plasmacytomas, and gain(1q21), potentially leading to evidence-based use in patients with these characteristics. Additional clinical data from larger patient populations will allow successful translation from current clinical study findings with anti-CD38 antibodies to informing real-world practice [54, 69–71, 83, 84].

Anti-CD38 antibodies mediate anti-myeloma activity through multiple mechanisms of action both in patients with RRMM and with newly diagnosed MM, although immunomodulatory effects may be greater in earlier disease settings in which the immune system has not been affected by prior treatments [85]. Results of phase III studies of daratumumab in combination with bortezomib-thalidomide-dexamethasone, bortezomib-melphalan-prednisone, or lenalidomide-dexamethasone and of isatuximab in combination with bortezomib-lenalidomide-dexamethasone have shown significant benefit from treatment with anti-CD38 combinations in transplant-eligible or -ineligible NDMM patients, compared with standard treatment [13, 14, 86]. Further results with isatuximab and daratumumab in NDMM with combination regimens such as bortezomib-lenalidomide-dexamethasone or carfilzomib-lenalidomide-dexamethasone are awaited with interest (NCT03319667, NCT03617731, NCT04483739, NCT03710603, NCT03652064).

Clinical experience with sequencing of anti-CD38 antibody therapies is currently limited to small numbers of patients; prospective studies of larger patient populations may contribute to establish criteria for optimal antibody selection and timing of treatment, as well as to define their overall efficacy, which in turn should meaningfully inform real world practice [84]. Similarly, further investigation may provide more direct evidence on their respective resistance profiles [87] and further insights to optimize anti-CD38 antibody therapeutic strategies for patients with RRMM and thus improve patient outcomes.

In conclusion, as two anti-CD38 monoclonal antibodies, isatuximab and daratumumab, are available for use in patients with RRMM, we have provided an overview of the available evidence on mechanisms of action, differences in regulatory labeling, and clinical results from recent RRMM clinical trials for both of these antibodies. The findings of our analyses reflect the ongoing acquisition of new data, which to date have not demonstrated an overall, statistically significant superiority for either of them, in the absence of a randomized, head-to-head study. Further investigations on their pharmacodynamic properties as well as their clinical activity will provide more insight on the contribution of anti-CD38 antibody therapy to the management of patients with RRMM.

## References

[1] Deaglio S, Vaisitti T, Billington R, Bergui L, Omede P, Genazzani AA (2007). CD38/CD19: a lipid raft-dependent signaling complex in human B cells. Blood.

[2] Deaglio S, Mehta K, Malavasi F (2001). Human CD38: a (r)evolutionary story of enzymes and receptors. Leuk Res.

[3] Liu Q, Kriksunov IA, Graeff R, Munshi C, Lee HC, Hao Q (2005). Crystal structure of human CD38 extracellular domain. Structure.

[4] Malavasi F, Deaglio S, Funaro A, Ferrero E, Horenstein AL, Ortolan E (2008). Evolution and function of the ADP ribosyl cyclase/CD38 gene family in physiology and pathology. Physiol Rev.

[5] van de Donk N, Richardson PG, Malavasi F (2018). CD38 antibodies in multiple myeloma: back to the future. Blood.

[6] Deaglio S, Morra M, Mallone R, Ausiello CM, Prager E, Garbarino G (1998). Human CD38 (ADP-ribosyl cyclase) is a counter-receptor of CD31, an Ig superfamily member. J Immunol.

[7] Munshi C, Aarhus R, Graeff R, Walseth TF, Levitt D, Lee HC (2000). Identification of the enzymatic active site of CD38 by site-directed mutagenesis. J Biol Chem.

[8] Lee HC, Walseth TF, Bratt GT, Hayes RN, Clapper DL (1989). Structural determination of a cyclic metabolite of NAD+ with intracellular Ca2+-mobilizing activity. J Biol Chem.

[9] Lee HC, Aarhus R, Levitt D (1994). The crystal structure of cyclic ADP-ribose. Nat Struct Biol.

[10] Chillemi A, Quarona V, Antonioli L, Ferrari D, Horenstein AL, Malavasi F (2017). Roles and modalities of ectonucleotidases in remodeling the multiple myeloma niche. Front Immunol.

[11] Horenstein AL, Faini AC, Morandi F, Bracci C, Lanza F, Giuliani N (2020). The circular life of human CD38: from basic science to clinics and back. Molecules.

[12] European Commission approves Sarclisa® (isatuximab) for adults with relapsed and refractory multiple myeloma. Press release. Sanofi; June 2, 2020. Accessed March 16, 2022. https://www.sanofi.com/en/media-room/press-releases/2020/2020-06-02-12-47-38

[13] Darzalex. Prescribing information. Janssen; March 2022. Accessed March 16, 2022. https://www.janssenlabels.com/package-insert/product-monograph/prescribing-information/DARZALEX-pi.pdf

[14] Darzalex Faspro. Prescribing information. Janssen; January 2022. Accessed March 16, 2022. https://www.janssenlabels.com/package-insert/product-monograph/prescribing-information/DARZALEX+Faspro-pi.pdf

[15] Sarclisa. Prescribing information. Sanofi; March 2021. Accessed March 16, 2022. https://products.sanofi.us/Sarclisa/sarclisa.pdf

[16] European Medicines Agency. Sarclisa, INN-Ixatuximab. Summary of product characteristics. 2021. Accessed March 16, 2022. https://www.ema.europa.eu/en/documents/product-information/sarclisa-epar-product-information_en.pdf

[17] European Commission grants marketing authorisation for Darzalex (daratumumab) subcutaneous formulation for all currently approved daratumumab intravenous formulation indications. Press release. Janssen; June 4, 2020. Accessed March 16, 2022. https://myelomabeacon.org/pr/2020/06/04/european-approval-subcutaneous-darzalex/

[18] Darzalex. European Medicines Agency. Updated August, 2020. Accessed March 16, 2022. https://www.ema.europa.eu/en/documents/overview/darzalex-epar-medicine-overview_en.pdf

[19] Martin TG, Corzo K, Chiron M, Velde HV, Abbadessa G, Campana F (2019). Therapeutic opportunities with pharmacological inhibition of CD38 with isatuximab. Cells.

[20] Wetzel M, Nicolazzi C, Cai T, Vallee F, Deckert J, Dumontet C et al (2013) Preclinical characterization of SAR650984, a humanized anti-CD38 antibody for the treatment of multiple myeloma. International Myeloma Workshops: Kyoto, Japan. P288.

[21] Deckert J, Wetzel MC, Bartle LM, Skaletskaya A, Goldmacher VS, Vallée F (2014). SAR650984, a novel humanized CD38-targeting antibody, demonstrates potent antitumor activity in models of multiple myeloma and other CD38+ hematologic malignancies. Clin Cancer Res.

[22] Lammerts van Bueren J, Jakobs D, Kaldenhoven N, Roza M, Hiddingh S, Meesters J (2014). Direct in vitro comparison of daratumumab with surrogate analogs of CD38 antibodies MOR03087, SAR650984 and Ab79. Blood.

[23] van de Donk NW, Janmaat ML, Mutis T, Lammerts van Bueren JJ, Ahmadi T, Sasser AK (2016). Monoclonal antibodies targeting CD38 in hematological malignancies and beyond. Immunol Rev.

[24] Moreno L, Perez C, Zabaleta A, Manrique I, Alignani D, Ajona D (2019). The mechanism of action of the anti-CD38 monoclonal antibody isatuximab in multiple myeloma. Clin Cancer Res.

[25] Horenstein AL, Chillemi A, Quarona V, Zito A, Roato I, Morandi F (2015). NAD^+^-metabolizing ectoenzymes in remodeling tumor-host interactions: the human myeloma model. Cells.

[26] Malavasi F, Faini A, Morandi F, Castella B, Incarnato D, Oliviero S (2021). Molecular dynamics of targeting CD38 in multiple myeloma. Br J Haematol.

[27] Overdijk MB, Jansen JH, Nederend M, Lammerts van Bueren JJ, Groen RW, Parren PW (2016). The therapeutic CD38 monoclonal antibody daratumumab induces programmed cell death via Fcγ receptor-mediated cross-linking. J Immunol.

[28] Kinder M, Bahlis N, Malavasi F, de Goeij B, Babich A, Sendecki J (2021). Comparison of CD38 antibodies in vitro and ex vivo mechanisms of action in multiple myeloma. Haematologica.

[29] Jiang H, Acharya C, An G, Zhong M, Feng X, Wang L (2016). SAR650984 directly induces multiple myeloma cell death via lysosomal-associated and apoptotic pathways, which is further enhanced by pomalidomide. Leukemia.

[30] de Weers M, Tai YT, van der Veer MS, Bakker JM, Vink T, Jacobs DC (2011). Daratumumab, a novel therapeutic human CD38 monoclonal antibody, induces killing of multiple myeloma and other hematological tumors. J Immunol.

[31] Feng X, Zhang L, Acharya C, An G, Wen K, Qiu L (2017). Targeting CD38 suppresses induction and function of T regulatory cells to mitigate immunosuppression in multiple myeloma. Clin Cancer Res.

[32] Zhu C, Song Z, Wang A, Srinivasan S, Yang G, Greco R (2020). Isatuximab acts through Fc-dependent, independent, and direct pathways to kill multiple myeloma cells. Front Immunol.

[33] Viola D, Dona A, Caserta E, Troadec E, Besi F, McDonald T (2021). Daratumumab induces mechanisms of immune activation through CD38+ NK cell targeting. Leukemia.

[34] Golay J, Taylor RP (2020). The role of complement in the mechanism of action of therapeutic anti-cancer mAbs. Antibodies (Basel).

[35] Nijhof IS, Casneuf T, van Velzen J, van Kessel B, Axel AE, Syed K (2016). CD38 expression and complement inhibitors affect response and resistance to daratumumab therapy in myeloma. Blood.

[36] Overdijk MB, Verploegen S, Bögels M, van Egmond M, Lammerts van Bueren JJ, Mutis T (2015). Antibody-mediated phagocytosis contributes to the anti-tumor activity of the therapeutic antibody daratumumab in lymphoma and multiple myeloma. MAbs.

[37] Neri P, Maity R, Tagoug I, McCulloch S, Duggan P, Jimenez-Zepeda V (2018). Immunome single cell profiling reveals T cell exhaustion with upregulation of checkpoint inhibitors LAG3 and Tigit on marrow infiltrating T lymphocytes in daratumumab and IMiDs resistant patients. Blood.

[38] Platzer B, Stout M, Fiebiger E (2014). Antigen cross-presentation of immune complexes. Front Immunol.

[39] Atanackovic D, Yousef S, Shorter C, Tantravahi SK, Steinbach M, Iglesias F (2020). In vivo vaccination effect in multiple myeloma patients treated with the monoclonal antibody isatuximab. Leukemia.

[40] Horenstein A, Quarona V, Toscani D, Costa F, Chillemi A, Pistoia V (2016). Adenosine generated in the bone marrow niche through a CD38-mediated pathway correlates with progression of human myeloma. Mol Med.

[41] Sitkovsky M, Ohta A (2013). Targeting the hypoxia-adenosinergic signaling pathway to improve the adoptive immunotherapy of cancer. J Mol Med (Berl).

[42] Calabretta E, Carlo-Stella C (2020). The many facets of CD38 in lymphoma: from tumor-microenvironment cell interactions to acquired resistance to immunotherapy. Cells.

[43] van de Donk N, Usmani SZ (2018). CD38 antibodies in multiple myeloma: mechanisms of action and modes of resistance. Front Immunol.

[44] Krejcik J, Casneuf T, Nijhof IS, Verbist B, Bald J, Plesner T (2016). Daratumumab depletes CD38+ immune regulatory cells, promotes T-cell expansion, and skews T-cell repertoire in multiple myeloma. Blood.

[45] Dimopoulos M, Bringhen S, Anttila P, Capra M, Cavo M, Cole C (2021). Isatuximab as monotherapy and combined with dexamethasone in patients with relapsed/refractory multiple myeloma. Blood.

[46] Richardson PG, Beksaç M, Špička I, Mikhael J (2020). Isatuximab for the treatment of relapsed/refractory multiple myeloma. Expert Opin Biol Ther.

[47] Chapuy CI, Nicholson RT, Aguad MD, Chapuy B, Laubach JP, Richardson PG (2015). Resolving the daratumumab interference with blood compatibility testing. Transfusion.

[48] Attal M, Richardson PG, Rajkumar SV, San-Miguel J, Beksac M, Spicka I (2019). Isatuximab plus pomalidomide and low-dose dexamethasone versus pomalidomide and low-dose dexamethasone in patients with relapsed and refractory multiple myeloma (ICARIA-MM): a randomised, multicentre, open-label, phase 3 study. Lancet.

[49] Song Z, Bedel O, Zhang B, Hopke J, Deng G, Macé S (2021). Anti-CD38 interference with blood compatibility testing: differentiating isatuximab and daratumumab via functional epitope mapping. Cancer Res.

[50] Lancman G, Arinsburg S, Jhang J, Cho HJ, Jagannath S, Madduri D (2018). Blood transfusion management for patients treated with anti-CD38 monoclonal antibodies. Front Immunol.

[51] Quach H, Benson S, Haysom H, Wilkes A, Zacher N, Cole-Sinclair M (2018). Considerations for pre-transfusion immunohaematology testing in patients receiving the anti-CD38 monoclonal antibody daratumumab for the treatment of multiple myeloma. Intern Med J.

[52] Izaguirre EC, Del Mar L-H, González LL, CA C,  (2020). New method for overcoming the interference produced by anti-CD38 monoclonal antibodies in compatibility testing. Blood Transfus.

[53] van de Donk NWOH, El Haddad O (2016). Interference of daratumumab in monitoring multiple myeloma patients using serum immunofixation electrophoresis can be abrogated using the daratumumab IFE reflex assay (DIRA). Clin Chem Lab Med.

[54] Moreau P, Dimopoulos MA, Mikhael J, Yong K, Capra M, Facon T (2021). Isatuximab, carfilzomib, and dexamethasone in relapsed multiple myeloma (IKEMA): a multicentre, open-label, randomised phase 3 trial. Lancet.

[55] Martin T, Mikhael J, Hajek R, Kim K, Suzuki K, Hulin C (2020). Depth of response and response kinetics of isatuximab plus carfilzomib and dexamethasone in relapsed multiple myeloma: IKEMA interim analysis. Blood.

[56] Murray D, Puig N, Kristinsson S, Usmani S, Dispenzieri A, Bianchi G (2021). Mass spectrometry for the evaluation of monoclonal proteins in multiple myeloma and related disorders: an International Myeloma Working Group Mass Spectrometry Committee Report. Blood Cancer J.

[57] Moreau P, Parmar G, Prince M, Ocio E, Karanes C, Madan S et al 2021 A multi-center, Phase 1b study to assess the safety, pharmacokinetics and efficacy of subcutaneous isatuximab plus pomalidomide and dexamethasone, in patients with relapsed/refractory multiple myeloma. International Myeloma Workshop - XVIII. Abstract P-207

[58] Usmani S, Karanes C, Bensinger W, D’Souza A, Raje N, Tuchman S (2021). Final results of a Phase 1b study of isatuximab short-duration fixed-volume infusion combination therapy for RRMM. Leukemia.

[59] Trakada G, Kastritis E, Gavriatopoulou M, Velentza L, Fotiou D, Ziogas DC (2019). Pulmonary function abnormalities are common in patients with multiple myeloma and are independently associated with worse outcome. Ann Hematol.

[60] Chari A, Suvannasankha A, Fay JW, Arnulf B, Kaufman JL, Ifthikharuddin JJ (2017). Daratumumab plus pomalidomide and dexamethasone in relapsed and/or refractory multiple myeloma. Blood.

[61] Dimopoulos M, Terpos E, Boccadoro M, Delimpasi S, Beksac M, Katodritou E (2021). Daratumumab plus pomalidomide and dexamethasone versus pomalidomide and dexamethasone alone in previously treated multiple myeloma (APOLLO): an open-label, randomised, phase 3 trial. Lancet Oncol.

[62] Study of carfilzomib, daratumumab and dexamethasone for patients with relapsed and/or refractory multiple myeloma (CANDOR). ClinicalTrials.gov identifier: NCT03158688. Accessed March 16, 2022. https://clinicaltrials.gov/ct2/show/NCT03158688

[63] Multinational clinical study comparing isatuximab, carfilzomib and dexamethasone to carfilzomib and dexamethasone in relapse and/or refractory multiple myeloma patients (IKEMA). ClinicalTrials.gov identifier: NCT03275285. Accessed March 16, 2022. https://clinicaltrials.gov/ct2/show/NCT03275285

[64] Moreau P, Dimopoulos MA, Mikhael J, Yong K, Capra M, Facon T et al 2020 Isatuximab plus carfilzomib and dexamethasone vs carfilzomib and dexamethasone in relapsed/refractory multiple myeloma (IKEMA): interim analysis of a phase 3, randomized, open-label study. European Hematology Association; June 14, 2020. Abstract LB2603

[65] Usmani SZ, Weiss BM, Plesner T, Bahlis NJ, Belch A, Lonial S (2016). Clinical efficacy of daratumumab monotherapy in patients with heavily pretreated relapsed or refractory multiple myeloma. Blood.

[66] Mikhael J, Richardson P, Usmani SZ, Raje N, Bensinger W, Karanes C (2019). A phase 1b study of isatuximab plus pomalidomide/dexamethasone in relapsed/refractory multiple myeloma. Blood.

[67] Offidani M, Corvatta L, Morè S, Nappi D, Martinelli G, Olivieri A (2021). Daratumumab for the management of newly diagnosed and relapsed/refractory multiple myeloma: current and emerging treatments. Front Oncol.

[68] APOLLO (MMY3013) Study. JanssenMD Professional Information Resource. December 18, 2020. Accessed August 31, 2021. https://www.janssenmd.com/darzalex-faspro/clinical-data/clinical-studies/apollo-mmy3013-study

[69] Beksac M, Richardson P, Unal A, Corradini P, DeLimpasi S, Gulbas Z et al 2020 Isatuximab plus pomalidomide and dexamethasone in patients with relapsed/refractory multiple myeloma and soft-tissue plasmacytomas: ICARIA-MM subgroup analysis. American Society of Hematology Annual Meeting 2020. Abstract 2289

[70] Richardson P, Harrison S, Facon T, Yong K, Raje N, Alegre A et al 2020 Isatuximab plus pomalidomide and dexamethasone in relapsed/refractory multiple myeloma patients with 1q21 gain: insights from phase 1 and phase 3 studies. Presented at 25th Annual Congress of European Hematology Association; June 11–14, 2020; Virtual. Accessed March 16, 2022. https://library.ehaweb.org/eha/2020/eha25th/293508

[71] Dimopoulos MA, Leleu X, Moreau P, Richardson PG, Liberati AM, Harrison SJ (2021). Isatuximab plus pomalidomide and dexamethasone in relapsed/refractory multiple myeloma patients with renal impairment: ICARIA-MM subgroup analysis. Leukemia.

[72] Martin T, Shah N, Richter J, Vesole D, Wong S, Huang C (2021). Phase 1b trial of isatuximab, an anti-CD38 monoclonal antibody, in combination with carfilzomib as treatment of relapsed/refractory multiple myeloma. Cancer.

[73] Moreau P, Dimopoulos MA, Mikhael J, Yong K, Capra M, Facon T (2022). Updated progression-free survival (PFS) and depth of response in IKEMA, a randomized phase III trial of isatuximab, carfilzomib and dexamethasone (Isa-Kd) vs Kd in relapsed multiple myeloma (MM). ESMO Virtual Plenary: VP5-2022. Ann Oncol.

[74] Chari A, Martinez-Lopez J, Mateos MV, Bladé J, Benboubker L, Oriol A (2019). Daratumumab plus carfilzomib and dexamethasone in patients with relapsed or refractory multiple myeloma. Blood.

[75] Dimopoulos M, Quach H, Mateos MV, Landgren O, Leleu X, Siegel D (2020). Carfilzomib, dexamethasone, and daratumumab versus carfilzomib and dexamethasone for patients with relapsed or refractory multiple myeloma (CANDOR): results from a randomised, multicentre, open-label, phase 3 study. Lancet.

[76] Usmani SZ, Quach H, Mateos MV, Landgren O, Leleu X, Siegel D (2022). Carfilzomib, dexamethasone, and daratumumab versus carfilzomib and dexamethasone for patients with relapsed or refractory multiple myeloma (CANDOR): updated outcomes from a randomised, multicentre, open-label, phase 3 study. Lancet Oncol.

[77] Mikhael J, Belhadj-Merzoug K, Hulin C (2021). A phase 2 study of isatuximab monotherapy in patients with multiple myeloma who are refractory to daratumumab. Blood Cancer J.

[78] Nijhof IS, Groen RW, Lokhorst HM, van Kessel B, Bloem AC, van Velzen J (2015). Upregulation of CD38 expression on multiple myeloma cells by all-trans retinoic acid improves the efficacy of daratumumab. Leukemia.

[79] Richardson P, Perrot A, San-Miguel J, Beksac M, Spicka I, Leleu X (2021). Updates from ICARIA-MM, a phase 3 study of isatuximab (Isa) plus pomalidomide and low-dose dexamethasone (Pd) versus Pd in relapsed and refractory multiple myeloma (RRMM). J Clin Oncol.

[80] Becnel M, Horowitz S, Thomas S, Iyer S, Patel K, Manasanch E (2020). Descriptive analysis of isatuximab use following prior daratumumab in patients with relapsed/refractory multiple myeloma. Blood.

[81] Franssen LE, Stege CAM, Zweegman S, van de Donk N, Nijhof IS (2020). Resistance mechanisms towards CD38-directed antibody therapy in multiple myeloma. J Clin Med.

[82] Saltarella I, Desantis V, Melaccio A, Solimando AG, Lamanuzzi A, Ria R (2020). Mechanisms of resistance to anti-CD38 daratumumab in multiple myeloma. Cells.

[83] Jelinek T, Sevcikova T, Popkova T, Cerna L, Broskevicova L, Brozova L et al (2020) Limited efficacy of daratumumab in multiple myeloma with extramedullary disease. European Haematology Association 2020; Abstract EP1030

[84] Richardson P, San Miguel J, Moreau P, Hajek R, Dimopoulos M, Laubach J (2018). Interpreting clinical trial data in multiple myeloma: translating findings to the real-world setting. Blood Cancer J.

[85] Tai Y-T, Anderson K (2016). A new era of immune therapy in multiple myeloma. Blood.

[86] Goldschmidt H, Mai E, Nievergall E, Fenk R, Bertsch U, Tichy D (2021). Addition of isatuximab to lenalidomide, bortezomib and dexamethasone as induction therapy for newly-diagnosed, transplant-eligible multiple myeloma patients: the phase III GMMG-HD7 trial. Blood.

[87] Plesner T, van de Donk N, Richardson PG (2020). Controversy in the use of CD38 antibody for treatment of myeloma: is high CD38 expression good or bad?. Cells.

